# Approach to the Patient With Turner Syndrome

**DOI:** 10.1210/clinem/dgaf517

**Published:** 2025-09-13

**Authors:** Helen E Turner, Emma B Johannsen, Arlene Smyth, Elizabeth Orchard, Claus H Gravholt

**Affiliations:** Department of Endocrinology, OCDEM, Churchill Hospital, Oxford OX3 7LE, UK; Department of Endocrinology and Internal Medicine, Aarhus University Hospital, Aarhus 8200, Denmark; Turner Syndrome Support Society Clydesbank, Glasgow G81 2NR, UK; Department of Cardiology, John Radcliffe Hospital, Oxford OX3 9DU, UK; Department of Endocrinology and Internal Medicine, Aarhus University Hospital, Aarhus 8200, Denmark

**Keywords:** Turner Syndrome, ovarian insufficiency, aortic dilatation, puberty, autoimmunity

## Abstract

Turner syndrome is diagnosed in a female individual with partial or complete loss of the second sex chromosome and is reported in 1 in 2000 to 1 in 2500 live births. Common features include short stature and ovarian dysgenesis; subsequent ovarian insufficiency leading to delayed/absent puberty and infertility in the majority. It is associated with increased morbidity and mortality, due to comorbidities occurring throughout the lifespan, including congenital and acquired cardiovascular abnormalities, autoimmune disease, osteoporosis and other skeletal abnormalities, and metabolic dysfunction as well as neurocognitive challenges. Management may involve coordination of several specialties in addition to patient/relative information and support. Treatment with growth hormone during childhood and adolescence and sex hormone replacement therapy forms the cornerstone of medical treatment. Recent review of evidence and development of recommendations inform a practical approach to management with an aim to reduce morbidity and thus improve outcomes in the future.

The aim of our “Approach” is to provide a practical approach to the key aspects of the lifetime follow-up of a patient with Turner syndrome (Fig. 1). Utilizing the recently published international practice guidelines on the care of women with Turner syndrome, we discuss the management of 3 patients. All 3 cases are based on clinic patients under current review and gave their consent for anonymized description in this Approach. Readers are therefore encouraged to refer to the referenced reviews/guidelines if more detailed background, evidence, and references are sought (1).

**Figure 1.**
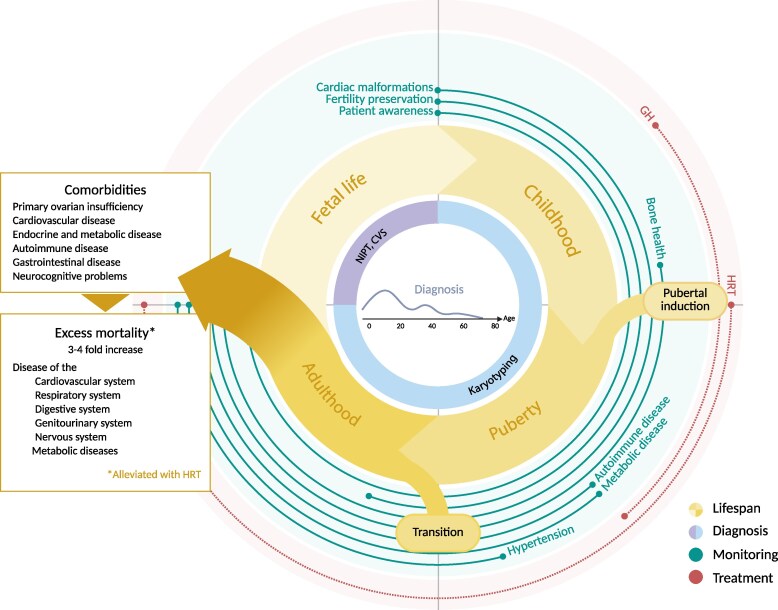
Lifespan of a person with Turner syndrome (TS). The central graph illustrates the typical age of diagnosis of TS and emphasizes that while diagnosis is commonly in childhood/adolescence, patients may be diagnosed later in life. The inner ring (blue) illustrates which methodology can lead to diagnosis. It includes noninvasive prenatal testing (NIPT) and chorionic villus sampling (CVS); however, in those individuals with prenatal diagnosis, postnatal confirmation check of karyotype should be performed in all individuals. The yellow ring is divided into 4 periods of life that need special attention in the care of an individual with TS. The green lines illustrate the typical time points when different comorbidities in TS can be diagnosed and when issues such as fertility and cardiovascular risk should be considered, and the red lines illustrate when GH and HRT may be relevant. To the left, boxes mention the most common comorbidities present in TS and frequent reasons for the excess mortality seen in TS.

## Case 1

A 17-year-old girl was referred to the adult endocrine clinic by her general practitioner (GP), due to primary amenorrhea and short stature. She had initially seen her GP at age 15 years and was reassured, but presented again at age 17 years. Initial primary care investigations showed follicle-stimulating hormone level of 89 IU/L, estradiol < 37 pmol/L and height of 152.7 cm (3.6 centile aged 18 years). On review, she had a past history of recurrent ear infections as a child, had always been shorter than girls in her class, and more recently noted multiple skin moles. She was breast stage IV and was below the third centile for height. Transabdominal ultrasound showed a prepubertal uterus and the ovaries were not visualized. Hand and wrist x-ray showed a significantly delayed bone age (13 years [chronological age 18 years]). Karyotype was consistent with Turner syndrome: 45,X [99/100]/46,XY [1/100].

Following discussion with the patient and family, she was referred for gynecological assessment in view of the risk of gonadal dysgenesis and subsequent malignancy; magnetic resonance imaging (MRI) of the pelvis confirmed the small uterus and bilateral streak ovaries with no suspicious findings, and she underwent bilateral laparoscopic gonadectomy (benign histology). Low-dose estrogen had already been commenced for pubertal induction, and growth hormone (GH) was commenced. Investigations for comorbidities associated with Turner syndrome showed duplex kidneys on ultrasound, perforated left ear drum with normal audiogram, no elevation of tissue transglutaminase (tTG) autoantibodies and normal thyrotropin (TSH), satisfactory metabolic screen except elevated transaminases with a negative liver screen, and normal Fibroscan and MRI liver. On cardiac review, her blood pressure (BP) was 90/64 mmHg, transthoracic echocardiogram showed a bicuspid aortic valve, with no coarctation of the aorta and satisfactory indexed measurements of the aortic diameter (aortic size index [ASI; aortic diameter divided by body surface area cm/m^2^], ascending aorta 13 mm/m^2^ and aortic sinus 16 mm/m^2^). Her ongoing management included GH replacement, with good catch-up growth, which was discontinued when velocity < 2 cm/year, with a final height of 160 cm. Uterine size improved from 2.9 cm to 6.3 cm with satisfactory breast development, and progesterone commenced when she started vaginal spotting (after 3 years of estrogen).

She was seen in the multidisciplinary transition clinic with endocrinology, clinical genetics, cardiology, and psychology and was enjoying her first year at university after a successful gap year traveling and planning a career in education. She is now receiving combined sequential transdermal hormone replacement, and fertility options have been discussed in outline.

This case highlights firstly the importance of accurate diagnosis of Turner syndrome. Turner syndrome is diagnosed in a phenotypic female with a karyotype which includes one intact X and complete/partial absence of a second sex chromosome (1) in conjunction with one or more clinical manifestations such as typical physical/structural features, short stature, ovarian insufficiency, and other associated comorbidities. There is, however, significant heterogeneity, and some individuals may have no obvious clinical abnormalities. Karyotype remains the gold standard examining at least 30 metaphases, notwithstanding newer techniques, and should be performed in a female individual with typical physical appearance, but also considered in individuals with short stature, ovarian insufficiency and infertility, and left sided congenital cardiac defects (1). However, it is important that the details of the actual karyotype are noted; presence of Y chromosome material occurs in approximately 10% of Turner syndrome and is associated with increased risk of gonadoblastoma, but low risk of malignancy (11%-20% incidence of gonadoblastoma 2–4). Moreover, while data are limited due to early gonadectomy, spontaneous puberty has been reported in 11% to 21.4% and spontaneous pregnancies occasionally reported in those with Y chromosome material (2, 3). While previous guidelines have recommended early prophylactic gonadectomy, the recent update recognizes patient autonomy and an individualized approach is recommended that weighs the risks of malignancy and potential benefit of maintained gonadal function while ensuring secure follow-up (1, 4).

Many studies have highlighted the association of particular karyotype with phenotype and this can be helpful in providing a guide to comorbidities (5, 6) (Table 1); however, the differing frequencies demonstrate that the relationship is not clear-cut and other more complex mechanisms and possible genetic pathways are likely to play a role (Table 2).

**Table 1.** Karyotype, frequency, and phenotypic association in Turner syndrome

Karyotype	Frequency, % (1)	Phenotypic association relative to other Turner syndrome karyotypes
45,X (Monosomy X)	40%-50%	↑Comorbidities ↑Congenital cardiac abnormalities ↑Mortality
45,X/46,XX (Mosaicism with 46,XX)	15%-25%	Milder phenotype ↑Spontaneous menarche and pregnancy
45,X/47,XXX or 45,X/46,XX/47,XXX (Mosaicism with 47,XXX)	3%	Milder phenotype ? ↑neurocognitive and mental health challenges
45,X/46,XY (Mosaicism with XY)	10%-12%	Risk of gonadoblastoma ↓Autoimmune disease ↓Coarctation
45,X/46,X,r(X) (Ring chromosome)	Rare	Phenotype depends on *XIST*; Loss of *XIST* ↑severe neurocognitive challenges No loss *XIST* ↑metabolic syndrome
46,X,i(Xq); 46,X,idic (Xp) (Isochromosome Xq)	15%	Intermediate phenotype
46,XX,del (p11) (Proximal deletion of Xp)		
X-autosomal trans, unbalanced		

**Table 2.** Putative effects of genetic changes on phenotype in Turner syndrome

Genetic change	Possible effect
Reduction in methylation status	Increase in mortality
Different levels of mosaicism in affected organs	Fertility in 45,X karyotype individual
PAR1 genes and X-Y gene pairs dose effect	Neuropsychological effects, metabolic effects, low-grade inflammatory condition
Second hit effect on autosomal gene in combination with haploinsufficiency of X chromosome	Bicuspid aortic valve and aortopathy (*TIMP1*/*TIMP3* alleles)
X chromosome dose effect	*ZFX* gene (possible effect on ovarian function and growth)

This case also illustrates the relatively late diagnosis of Turner syndrome, which is not infrequent. The median age of diagnosis remains around age 15, with an earlier peak due to phenotypic appearance, the diagnosis of congenital cardiac abnormalities, or reduced growth, and a later peak when adolescents are noted to fail to proceed through normal puberty, with a later range of age of diagnosis up to postmenopausal age (Fig. 1), the latter potentially missing out on optimal GH/estradiol replacement therapy and management of associated comorbidities (7–9). A recent UK Biobank study of 245 000 individuals detected 30 women with undiagnosed 45,X karyotype, and a larger number with mosaicism fulfilling diagnostic criteria but mild phenotype, highlighting the importance of improving diagnosis (10). In the future, clinical feature-based algorithms recognizing features such as short stature, recurrent ear infections, and structural abnormalities (1) as well as internet-based search engine approaches for patients may improve diagnosis (eg, Missing an X | Turner Syndrome Information). Neonatal screening has also been proposed as a means of securing early diagnosis in all (1, 11).

Poor growth and short stature is common in girls with Turner syndrome (related to absence of the short stature homeobox-containing gene (*SHOX*) in the pseudo-autosomal region of the X chromosome, IGF-1 resistance, and lack of pubertal growth spurt with estradiol deficiency); thus, GH replacement is important as highlighted in the case. The average adult final height deficit for individuals with Turner syndrome is approximately 20 cm and is usually disproportionate (shorter limbs than trunk) as well as associated with skeletal abnormalities such as scoliosis, Madelung deformity, and cubitus and genu valgum (12, 13). Recent data, although limited with respect specifically to individuals with Turner syndrome, are largely reassuring in terms of malignancy, scoliosis, and metabolic outcomes, but more data are needed regarding impact on aortic risk (1, 14–16).

The majority of individuals with Turner syndrome will require estrogen replacement (Fig. 1); approximately 20% will go through spontaneous puberty, but subsequent premature ovarian insufficiency is common (17). Pubertal induction with low-dose estrogen is initiated at age 11 or 12 if a diagnosis of Turner syndrome is confirmed; however, later diagnosis, as aforementioned, is common, and initiation of GH treatment is a reasonable approach if there is remaining growth potential, as in case 1. Lower doses of estrogen will maximize growth potential in this later diagnostic group of patients with no fusion of the epiphyses and delayed bone age. Progestogen is added once breakthrough bleeding starts, and a cyclical pattern recommended (1, 18).

Transdermal 17 beta-estradiol is recommended, as it is physiological and effective in improving uterine growth, bone density, and optimizing metabolic outcomes (18, 19). Patient compliance and preference, however, is essential as the ultimate goal of adequate long-term hormone replacement therapy is to improve outcomes (20–22). The updated guidelines emphasize the importance of assessment of adequacy, including the development of secondary sexual characteristics, uterine growth during puberty, and bone health, by measurement of estradiol levels (adult suggested target 100-150 pg/mL [367-550 pmol/L]) (1, 23). Micronized progestogen is suggested as first-line replacement, although the higher potency of medroxyprogesterone may be helpful if there are bleeding problems or the use of an intra-uterine device. Higher dosing may be required to ensure adequate replacement, and corresponding increase in progestogen recommended.

While androgen levels are reduced in women with Turner syndrome, there are insufficient data to make recommendations regarding replacement (24).

Historically, the transition from the pediatric service to adult care has been a time when many individuals with Turner syndrome have been lost to follow-up (25). Thus, it is important to emphasize the importance of long-term follow-up at the time of transition. There are recommendations regarding tools to assess readiness for transition, evaluate the autonomy and understanding of the adolescent, discuss and revisit issues such as fertility and psychological concerns, and to introduce the family to the adult provider (26, 27). While there are useful tools to improve management and a gradual transition period is recommended, there is a recognition that this is dependent on resources and personnel (1).

Comorbidities are an important cause of the increased morbidity seen in girls and women with Turner syndrome; assessment and ongoing monitoring and management are essential for the long-term care of every individual with Turner syndrome (Table 3) and thus comprise an essential part of the long-term follow-up of any individual with Turner syndrome.

**Table 3.** Comorbidities associated with Turner syndrome

System	Comorbidity and approximate frequency %	Monitoring
Eyes	Strabismus 25% Refractive errors 40% Ptosis and prominent epicanthic folds	Comprehensive ophthalmological examination +/− FU
Ears	Hearing loss and ear infections 35-fold higher in TS Hearing loss 36%-84% ? Associated poor balance (vestibular)	Management of ear infections and complications Hearing screen at diagnosis, 2-3 y in childhood and every 5 y in adult +/− suspected Hearing aids/cochlear implants for sensorineural hearing loss Counsel regarding falls (particularly if low BMD)
Teeth	Increased dental problems (multifactorial) Increased incidence obstructive sleep apnea (craniofacial abnormalities, and increased BMI)	Annual dental assessment (particularly important if craniofacial abnormalities/congenital cardiac abnormalities) Assessment/sleep studies where suspected
Skin/lymphoedema	Lymphoedema 12%-27% Allergic/autoimmune conditions are commoner (dermatitis, eczema, psoriasis, vitiligo, alopecia areata and lichen sclerosis) Possible increased risk of melanoma/benign skin neoplasms	Often resolves in infancy Compression garments and lymphatic massage Annual skin examination and education of patients to monitor and report +/− referral
Renal structural abnormalities	Congenital structural abnormalities of the kidney and urinary tract 18%-60% (horseshoe kidney, duplicate collecting system 15%-20%, rotation, single kidney, multicystic kidneys)	Renal ultrasound at diagnosis Annual analysis for proteinuria if horseshoe kidney/renal agenesis; prompt treatment of infections, and assessment of renal function and hypertension +/− nephrology or urology referral where required
Metabolic disorders	Overweight/Obesity 50% Elevated lipids 30% adults Diabetes mellitus 25%-70% prevalence (T2 > T1 and possible TS specific);	Promote healthy lifestyle and exercise Screen lipid profile according to national guidelines/transition; abnormalities managed as per population guidelines Screen for diabetes mellitus; HbA1c or fasting glucose annually from age 10-12 or if symptoms; check autoantibodies to differentiate T1/2
Liver disease	Abnormal liver function tests (40%-80%) MASLD commonest Autoimmune	Annual LFT from age 10 y; persistent abnormalities investigated with liver screen, USS and in adults noninvasive assessment of severity (Fib-4, Fibroscan) +/− hepatology referral and healthy lifestyle (weight loss, avoid alcohol, exercise and optimize metabolic risk) HRT continued
Gastrointestinal disorders	↑Iron-deficiency anemia (IRR 3) and gastrointestinal hemorrhage (IRR 3) ↑Inflammatory bowel disease	Annual complete blood count +/− investigation of anemia
Autoimmune disorders	61% lifetime prevalence autoimmune disease Primary hypothyroidism Celiac disease (4%-7%) Vitamin B12 deficiency, vitiligo, T1 diabetes mellitus, Addison's disease, psoriasis, lichen sclerosis etc.	Annual/biannual TSH from diagnosis (+/− antibody screen) Coeliac autoantibody screen 2-5 y from diagnosis +/− features Screen if indicated
Bone and skeleton	Osteoporosis 25% with increased risk of fracture Skeletal abnormalities include scoliosis, genu valgum, and Madelung deformity	Optimal HRT, biannual vitamin D assessment +/− replacement DEXA after completion of growth and every 5-10 y (risk dependent) Physical examination at diagnosis +/− orthopedic management if required
Neoplasia	Inconsistent data (no increased risk/slight increase) with risk of bias	Adhere to general population cancer screening guidelines Awareness of incidental abnormalities

Comorbidities associated with Turner syndrome (TS), and their approximate frequency in individuals with TS, as well as suggestions for clinic monitoring (based on International Guidelines for Management of Turner Syndrome (1)). Cardiovascular risk monitoring is summarized in Fig. 5. Neurocognitive challenges are frequent in individuals with TS, are variable and complex and discussed in the text with the recommendation for assessment during clinic review.

Abbreviations: BMD, bone mineral density; BMI, body mass index; HRT, hormone replacement therapy; IRR, incidence risk ratio; LFT, liver function testing; MASLD, metabolic-associated steatotic liver disease; TSH, thyrotropin (thyroid stimulating hormone).

As highlighted in the case above, liver biochemical abnormalities are common in Turner syndrome. Two recent single-center comprehensive studies report a prevalence of 40% to 50% (28, 29), thus confirming population studies showing increased liver disease, cirrhosis, and liver-related mortality in individuals with Turner syndrome compared with the background population (30, 31). Persistent abnormalities should be investigated, and screening from age 10 onward is recommended (32). While metabolic-associated steatotic liver disease (MASLD) is common in this population, it is important to perform liver blood screen for hemochromatosis, autoimmune liver disease, and hepatitis, and imaging with ultrasound (for gall stones, focal lesions) to exclude other possible causes. Noninvasive risk assessment of liver damage using biochemical markers such as FIB-4 or ELF, and vibration-controlled transient elastography (Fibroscan) assessment of liver stiffness are helpful in assessing the individual risk of fibrosis; the aforementioned studies highlight a smaller proportion of individuals with Turner syndrome who demonstrate increased risk of fibrosis, and hence requirement for liver biopsy. Although unvalidated in younger women, MRI can be helpful (33). Management of MASLD includes advice regarding healthy lifestyle, weight loss, and optimization of vascular risk factors such as diabetes mellitus, hyperlipidemia, and hypertension, while newer therapies are under investigation in the wider context of MASLD (34, 35).

For each of the patients included in this Approach, care of their mental health and wellbeing, and neurocognition, should be assessed during clinic review and managed throughout the lifespan (for further extensive discussion and guidance see (1)). We would like to emphasize that although neurocognitive aspects of behavior may seem outside the usual area of interest for endocrinologists, it is important to have at least some understanding of these aspects when caring for female patients with Turner syndrome.

In childhood, developmental and learning challenges are frequent, and early recognition, as well as introduction of individualized care plans and educational support may be required. Adolescence and transition may be associated with additional challenges, such as fertility, body image, and confidence, and psychological support is crucial. During adulthood, depression, anxiety, and difficulties with social interaction are frequent, in addition to frequent complaints of fatigue and stress, compounded by organizational challenges as well as practical issues with employment, relationships (social and intimate), and for some individuals, problems of social isolation and difficulties with day-to-day independence (1, 36).

## Case 2

A 30-year-old patient was referred for ongoing management of Turner syndrome (karyotype 46,X,Xiq). Inflammatory bowel disease was diagnosed when she was aged 25 and required subtotal colectomy and ileostomy but had subsequently been stable. She was receiving combined sequential transdermal hormone replacement, and she was considering adoption since a single attempt at donor egg donation in vitro fertilization (IVF) had been unsuccessful.

**Figure 2.**
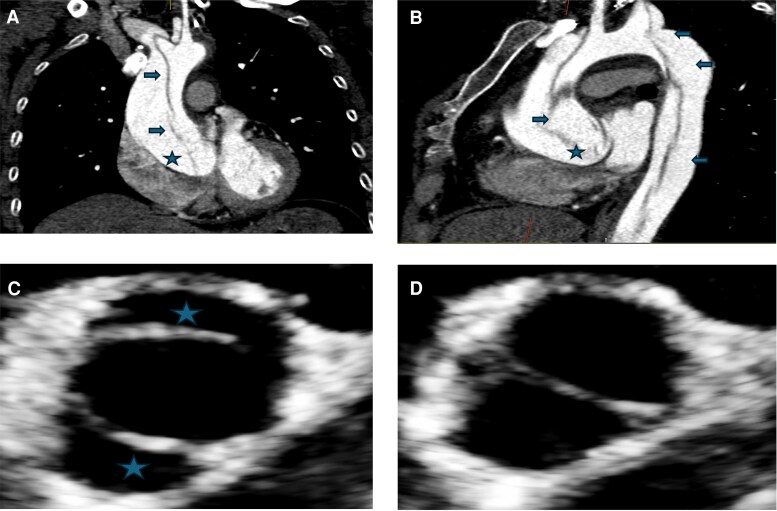
Cardiac imaging in Turner syndrome. A and B, Computed tomography scans of ascending and descending aorta, demonstrating dilated ascending aorta with dissection flap, marked with arrow extending from dilated aortic root, marked with star, through ascending aorta to descending aorta. C and D, Transthoracic echo demonstrating type 1 bicuspid aortic valve; panel C is open valve and panel D is closed valve. Anterior-posterior leaflets are marked with a star.

**Figure 3.**
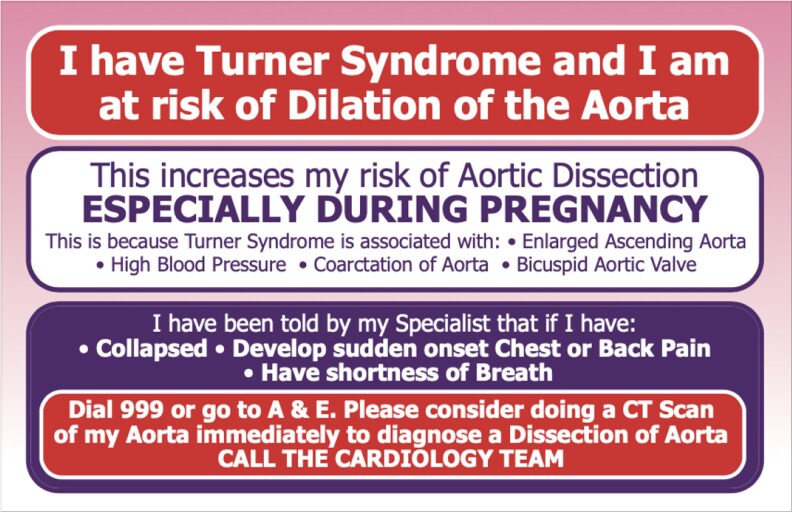
Cardiac alert card. Wallet-sized card for patients to carry, highlighting symptoms of concern for aortic dissection and action to take, to show to medical personnel in an emergency (locally developed and supported by Turner Syndrome Support Society, with permission) (37).

She was known to have hypertension, treated since age 28, and she had a bicuspid aortic valve (Fig. 2) with no aortic coarctation but a dilated aorta. Cardiac MRI performed after unsuccessful IVF showed a significant increase in the ascending aorta with maximum dimension 41 mm (normal < 32 mm) (increase 4 mm in 5 years [ASI, aortic sinus 27 mm/m^2^, ascending aorta 22 mm/m^2^]). Antihypertensive therapy was optimized (BP < 130/80 mmHg on ramipril and amlodipine), and urgent multidisciplinary discussion agreed that she required aortic root and ascending aortic replacement. The guidelines would classify her as high risk of aortic dissection. She was counseled on symptoms of acute aortic dissection; advised not to lift heavy loads at work and given an information card about aortic dissection and emergency action to take if symptoms arose (Fig. 3) (37) A couple of weeks prior to her planned elective aortic root repair, she developed typical symptoms of dissection while walking; her husband called the emergency services as instructed, showed the card and she had an emergency computed tomography scan (Fig. 2) and successful ascending aortic replacement on that day. She remains well 8 years post-dissection.

This case emphasizes that pre-pregnancy assessment and management of cardiovascular risk are very important aspects of any approach to caring for an individual with Turner syndrome.

Spontaneous menarche is reported in 8% of girls with 45,X karyotype, 52% with 45,X/46,XX karyotype, and 15% of other karyotypes (reviewed in (38)). Moreover, even in those with spontaneous menarche, pregnancy rates are low due to subsequent early premature ovarian insufficiency (and increased risk of miscarriage in Turner syndrome); spontaneous pregnancies are reported in 1% to 3% of those with 45,X karyotype (occasional pregnancies presumably due to ovarian mosaicism (8, 38)), 4% to 9% of those with structural abnormalities of the X chromosome, and 15% to 50% those with the milder phenotype usually associated with 45,X/46,XX karyotype (8, 38, 39).

**Figure 4.**
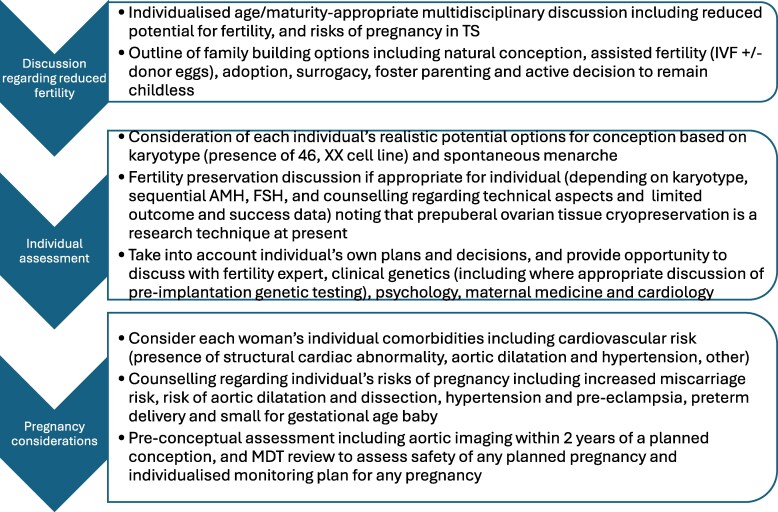
Approach to discussion of fertility and pregnancy when reviewing a patient with Turner syndrome (1, 38).

These cases illustrate the importance of early counseling regarding all parenting options, including the active decision to remain childless (1) (Fig. 4). Challenges regarding pregnancy, and reduced likelihood of fertility, comprise a very important aspect of psychological concerns for every individual (40, 41). Oocyte donation using donor eggs are an option for many women with Turner syndrome (from an unrelated or intrafamily donor), although pregnancy complications are higher than for other women with spontaneous pregnancies and IVF with autologous eggs (38, 39, 42); alternatives include adoption, surrogacy, and fostering (8).

In addition to this counseling, consideration of fertility preservation is important (43). Ovarian follicles are present in up to one-third of prepubertal girls, although this is most likely in those with the less severe phenotype associated with 46,XX mosaicism, and those with higher anti-Mullerian hormone. However, early premature ovarian insufficiency occurs commonly in those who have not required pubertal induction (44). At present, oocyte cryopreservation has been performed in a small number of individuals with Turner syndrome, requiring ovarian hyperstimulation and the relatively invasive procedure of egg retrieval; however, the results are very limited (summarized in (38)). Thus, individual assessment of each case, considering fertility potential, and counseling about the likelihood of success, are critical. In view of the early postnatal decline in follicles, early ovarian tissue cryopreservation is a potential future option in a prepubertal individual. However, this remains a field of ongoing research. Those individuals and families involved in such trials are counseled on the lack of any reported live birth following this technique, except for a single reported pregnancy (38, 45).

In addition to the challenges surrounding fertility, pregnancy in a woman with Turner syndrome, whether assisted or natural conception, is associated with increased risks compared to the background female population. The miscarriage rate is higher in Turner syndrome, and there is a reported increased risk of fetal abnormalities. Maternal hypertension in pregnancy is more common, with an increased risk of preterm delivery, and small for gestational age infant (9, 38, 46, 47). A major consideration is the risk of aortic dilatation, due to the increased cardiovascular stress of pregnancy superimposed on the increased risk of aortic dilatation and dissection in Turner syndrome. It is essential to assess women before conception, with up-to-date imaging (current guidance is transthoracic echo/cardiac MRI within 2 years of pregnancy), to evaluate the individual circumstances and concern (1). Interventions to reduce risk during a pregnancy include improving BP and diabetes management, aiming for BP < 130/80 mmHg. Low-dose aspirin can be used to reduce the danger of pre-eclampsia in those at higher risk. Frequent imaging of the woman's aorta should be undertaken in pregnancy; guidelines suggest that in the presence of aortic dilation (AHI > 20 mm/m, ASI >2.0 cm/m, or *Z* > 2.5) or at least one other risk factor (bicuspid aortic valve, aortic coarctation, hypertension, rapid increase in aortic diameter), transthoracic echocardiography should be performed at least once every 12 weeks during pregnancy (1). If there is a concern that the aorta has increased in size during pregnancy, MRI without contrast should be undertaken.

It is, however, to be noted that the historically poor outcomes for pregnancy in women with Turner syndrome have improved, with increased awareness of the importance of careful assessment, multidisciplinary management, counseling on alternatives where risk is significant, and adherence to expert recommendations (46, 48).

Cardiovascular disease accounts for 40% excess mortality in Turner syndrome, with increased incidence of congenital cardiac abnormalities, aortic dilation and dissection, and acquired vascular disease compared with the background population studies (9, 21, 31, 49). Thus, long-term careful management of cardiac abnormalities and vascular risk including the increased incidence of hypertension is essential for every girl and woman with Turner syndrome.

The commonest structural cardiac abnormality is a BAV (Fig. 2) which is reported in 25% patients, and coarctation of the aorta occurs in approximately 10% (50–52). Both these abnormalities in association with other less common aortic arch anomalies are associated with increased risk of aortic dilatation. In addition, anomalous venous drainage (15%-25% patients) may lead to right ventricular dilation and in a small proportion of patients to pulmonary hypertension (49–52).

Aortic dissection is commoner in Turner syndrome (160/100 000 compared with 6/100 000 person-years in the background population) and occurs at a significantly younger age (35 years) (53, 54). While multifactorial due to underlying ill-understood aortopathy, this is compounded by structural abnormalities such as bicuspid aorta and coarctation of the aorta, and hypertension (37, 53, 54). Individualized assessment of risk of dissection is recommended, based on assessment of aortic parameters which consider the often different body morphology and size in Turner syndrome (as standard thresholds for intervention would delay diagnosis of aortic dilatation and thus increase risk) (55). Therefore, indexing of the ascending aortic measurements (commonest site of dissection in Turner syndrome) using Z score (number of SD from the population mean), AHI (aortic diameters divided by body length [mm/m]) and ASI (aortic diameter divided by body surface area [cm/m^2^]) is important. ASI and Z score are predictive only if the body surface area is between 1 and 2 SD of the population mean, and limited data suggest that AHI may be more predictive (1). All individuals with Turner syndrome, but particularly those with additional risk factors for aortic dilatation (BAV, coarctation of aorta, rapid rate of aortic dilation (>3 mm/year), and hypertension) should be warned regarding the symptoms of aortic dissection in order that they can be aware to seek emergency medical care. The patient in case 2 had received a locally developed aortic alert card (supported by Turner Syndrome Support Society) and utilized this for urgent prompt review and appropriate management (Fig. 3).

In those with aortic disease (defined as Z > 2.5, AHI > 20 mm/m, ASI > 2 cm/m^2^), BAV, coarctation, or aortic dilation, scrupulous control of hypertension is essential, ideally using a beta-blocker, an angiotensin receptor blocker, or both (1, 55).

**Figure 5.**
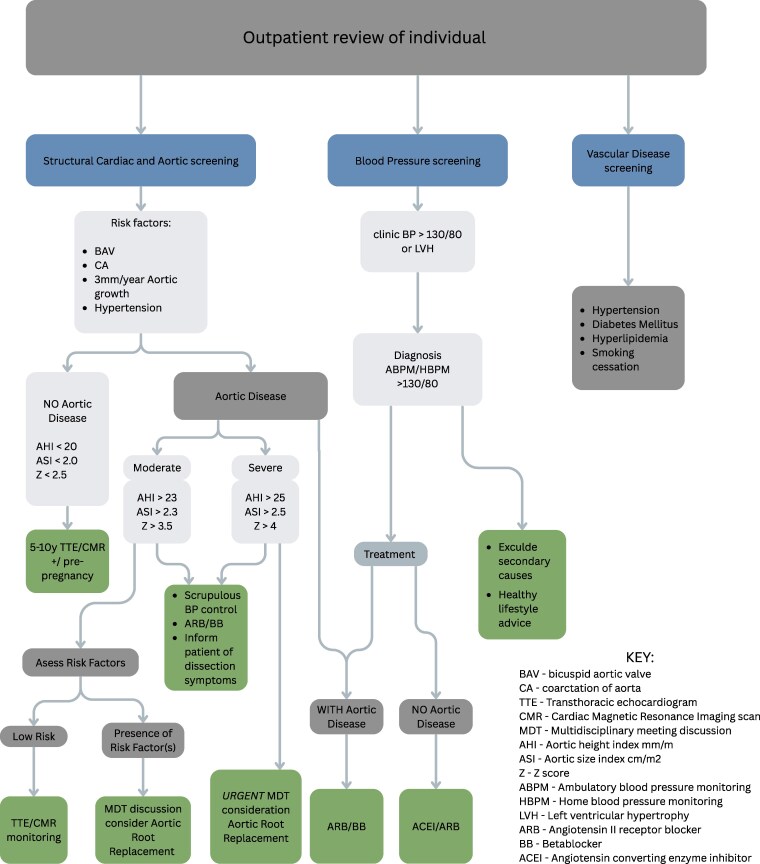
Summary patient cardiovascular assessment and management pathway to consider in every individual with Turner syndrome. Abbreviations: ABPM, ambulatory blood pressure monitoring; ACEI, angiotensin-converting enzyme inhibitor; AHI, aortic height index (mm/m); ARB, angiotensin II receptor blocker; ASI, aortic size index (cm/m^2^); BAV, bicuspid aortic valve; BB, beta-blocker; CA, coarctation of the aorta; HBPM, home blood pressure monitoring; LVH, left ventricular hypertrophy; MDT, multidisciplinary team discussion; TTE, transthoracic echocardiogram; Z, Z score.

The current guidelines recommend that those individuals with risks (BAV, coarctation, hypertension, rapid increase in aortic diameter [> 3 mm/year]) and moderate aortic dilation (AHI > 23 mm/m, ASI > 2.3 cm/m^2^ or Z > 3.5) should be discussed at the cardiothoracic multidisciplinary team meeting and counseled regarding symptoms of aortic dissection. Women with severe aortic dilation (notwithstanding any risk factors) defined as AHI > 23 mm/m, ASI >2.5 cm/m^2^ or Z > 4, should be considered for urgent aortic surgery (Fig. 5). Children with Turner syndrome are at lower risk of dissection and separate guidelines exist (1).

Hypertension often arises at a young age in individuals with Turner syndrome, and is 3- to 4-times more common in Turner syndrome than in the background population (56–58). Annual assessment of BP is recommended; and in those with suspected hypertension in clinic, further confirmation with ambulatory BP monitoring (ABPM) should be undertaken. This is also advantageous in detecting non-dipping nocturnal hypertension (reported in up to 50% of individuals with Turner syndrome (59, 60)). Recognizing that this is resource dependent, an alternative approach is patient home BP monitoring or exercise testing (1). Secondary causes of hypertension should be sought and treated, and lifestyle advice is important (1, 58). However, recognizing the lack of specific guidance, the recent guidelines suggest a diagnostic cutoff of 130/80 mmHg for screening and diagnosing hypertension. In the absence of aortic disease (see above) treatment with angiotensin-converting enzyme inhibitor or angiotensin receptor blocker is recommended (1). With current guidelines in place (Fig. 5), and the emphasis on risk management it should be possible to improve the early morbidity and mortality in Turner syndrome.

## Case 3

A woman with 45,X Turner syndrome was under regular clinical review since childhood. She was diagnosed with Turner syndrome at the age of 10 years and started GH treatment from 10 to 14 years of age. Puberty was induced from age 14 years due to primary ovarian insufficiency, and she developed primary hypothyroidism at the age of 18 years. She had an uneventful early adulthood, was normal weight, and was working as a preschool leader, became a diving instructor, and enjoyed mountain climbing. Later she became a body therapist and opened her own thriving business. She got married, and at the age of 40 years she underwent successful egg donation fertility treatment, followed by a successful and uneventful pregnancy (cesarean delivery) and had a normal boy. During her entire life she always had what she was told was psoriasis treated with topical glucocorticoids when necessary.

At the age of 51 years, she developed type 2 diabetes (no positive autoantibodies for type 1 diabetes). At the time of diagnosis, she weighed 58 kg (body mass index [BMI] 27.59); at the same time, she developed angina when jogging. She had never smoked and had normal lipids. She was referred to the cardiologists, where angiography demonstrated stenosis in her left anterior descending artery, but it was not possible to insert a stent due to the vascular anatomy. She was commenced on metformin, atorvastatin, and clopidogrel (she was intolerant of aspirin). However, treatment was complicated by severe (hospitalized on 2 occasions) exfoliative dermatitis requiring high dose topical glucocorticoids. The initial diagnosis of psoriasis was revised to pityriasis rubra pilaris and she is treated with methotrexate immunosuppression. Hypertension was diagnosed at the age of 52 years, and she is currently being treated with metformin, semaglutide, methotrexate, bisoprolol, pasugrel (anti-thrombotic), topical steroids, oral hormone replacement therapy (she cannot tolerate transdermal hormone replacement because of her skin condition) and levothyroxine. She has successfully lost weight and now has a BMI of 25.68.

This case illustrates the very frequent occurrence of the metabolic syndrome and development of type 2 diabetes (61, 62), even with only minimal weight gain in women with Turner syndrome. Adequate hormone replacement has been shown likely to reduce the risk of developing diabetes (61). The increased morbidity and mortality associated with cardiovascular and cerebrovascular disease in Turner syndrome is likely multifactorial, and therefore it is very important to pay careful attention to vascular risk factors during clinic review, including BP control, metabolic factors such as diabetes mellitus, hyperlipidemia, and MASLD, as well as advocating healthy lifestyle with weight management, exercise, and avoidance of smoking.

**Figure 6.**
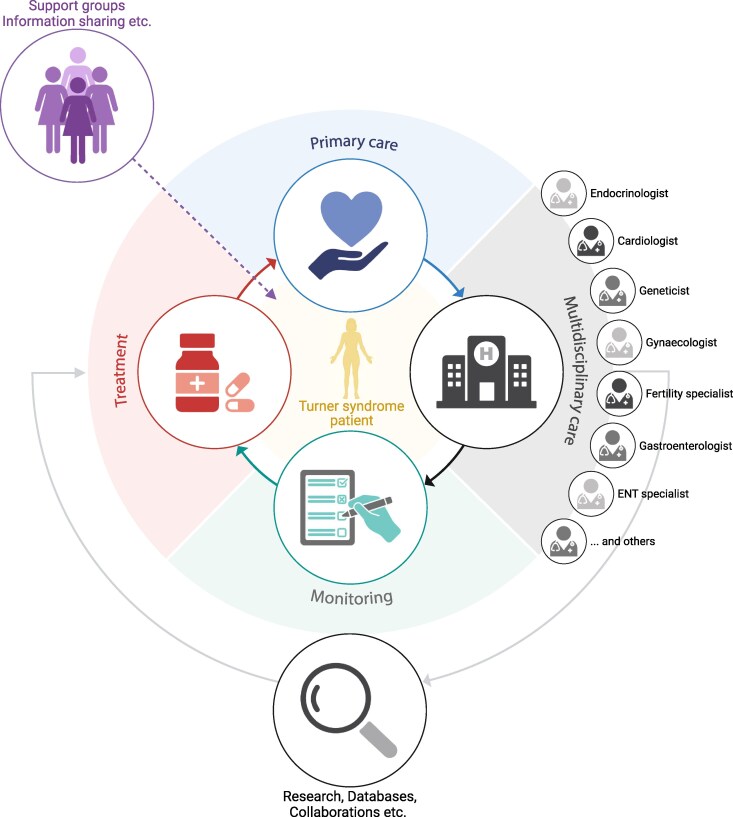
Multidisciplinary management of patients with Turner syndrome (TS). The figure depicts a model for care of an individual with TS; the patient is central with hospital monitoring (vigilant routine and “ad hoc” for management of intercurrent morbidity) and provision of advice regarding treatment. Importantly, it includes ongoing research and databases in order to optimize longer term outcomes.

These 3 cases highlight the importance of multidisciplinary care, and maintaining awareness of the various aspects of the long-term assessment and management which may need addressing at different time points during the lifespan of every individual with Turner syndrome (Fig. 6)

Patient feedback suggests the importance of a key central physician and an awareness of the time/resource commitment of attendance for different specialist investigations/review (63). Patient support groups provide very helpful written resources on both practical and medical aspects relevant for the whole lifespan of an individual as well as for the parent/partner and also employer/teacher (where appropriate) which may improve long-term outcomes (64). Patient support groups organize and coordinate local and national peer group meetings, where experiences can be shared, information disseminated, and advocacy for change provided. Current guidelines include recommending signposting of such organizations and provision of resources to patients; a patient/relative friendly publication was produced alongside the international guidelines and provides a comprehensive guide and reference for patients (turnersyndrome.org/_files/ugd/ff2c76_61e89cc2e343435488b65087e63721c0.pdf).

While strides have been made to improve care for each individual with Turner syndrome, and the comprehensive information utilized in the guidelines provide an invaluable aid to an approach for each patient's management, it should be recognized that these are *recommendations*; they represent a consensus agreement rather than necessarily total agreement, and they are *international*. Thus, perspectives may vary depending on resources/healthcare organization; moreover, for every individual in the clinic, patient autonomy is essential to respect. Furthermore, several of the recommendations are extrapolated on best available evidence where there may be none/expert opinion or extrapolation from other conditions.

Looking forward, there are areas which need further consideration in the future, as patient morbidity and mortality from congenital complications and adult comorbidities improve. The implications of recommendations need careful thought and resource/practicality/ethical consideration; for example, cardiac surgery may carry an excess risk in patients with Turner syndrome (65, 66), infection is a common problem highlighted in epidemiological studies (67), and gonadectomy/fertility preservation and autonomy need careful age-appropriate individual counseling (38). Vascular risk in older women, hearing loss, and balance issues may compound fracture risk, and social isolation may increase mental health challenges. Diversity and ethnic/religious considerations may require different approaches to early diagnosis and fertility management/family building options.

Finally, a better understanding of genetic abnormalities and associated phenotypic changes (21) may allow better delineation of individual risk and thus appropriately direct the multidisciplinary approach to clinical care to optimize outcomes and potentially reduce the increased morbidity and mortality associated with Turner syndrome.

## Data Availability

No new data were generated or analysed in support of this research.

## Abbreviations

AHI: aortic height index (aortic diameters divided by body length)
ASI: aortic size index (aortic diameter divided by body surface area)
GH: growth hormone
IVF: in vitro fertilization
MASLD: metabolic dysfunction–associated steatotic liver disease
MRI: magnetic resonance imaging
TSH: thyrotropin (thyroid stimulating hormone)
